# Tumor Site- and Stage-Specific Associations between Allelic Variants of Glutathione S-Transferase and DNA-Repair Genes and Overall Survival in Colorectal Cancer Patients Receiving 5-Fluorouracil-Based Chemotherapy

**DOI:** 10.1371/journal.pone.0069039

**Published:** 2013-07-23

**Authors:** Ching-Yu Lai, Ling-Ling Hsieh, Fung-Chang Sung, Reiping Tang, Chyi-Huey Bai, Fang-Yang Wu, Hung-Yi Chiou, Chih-Ching Yeh

**Affiliations:** 1 Department of Public Health, College of Public Health, China Medical University, Taichung City, Taiwan; 2 Department of Public Health, Chang Gung University, Guieshan, Taoyuan County, Taiwan; 3 Colorectal Section, Chang Gung Memorial Hospital, Guieshan, Taoyuan County, Taiwan; 4 School of Public Health, College of Public Health and Nutrition, Taipei Medical University, Taipei City, Taiwan; 5 Center of Excellence for Cancer Research, Taipei Medical University, Taipei City, Taiwan; MOE Key Laboratory of Environment and Health, School of Public Health, Tongji Medical College, Huazhong University of Science and Technology, China

## Abstract

**Introduction:**

Our retrospective cohort study investigated the effect of tumor site and stage on the associations between the allelic variants of glutathione S-transferase (GST) and DNA-repair genes and overall survival (OS) in CRC patients treated with 5-fluorouracil (5-FU)-based adjuvant chemotherapy.

**Material and Methods:**

We genotyped GSTM1, GSTT1, GSTP1 Ile105Val, XRCC1 Arg399Gln, XRCC3 Thr241Met, and XPD Lys751Gln in 491 CRC patients between 1995 and 2001. A Cox proportional-hazards model was used to calculate the hazard ratios (HRs) and 95% confidence intervals (CIs) for the relationships between the allelic variants and OS. Survival analyses were performed for each allelic variant by using the log-rank test and Kaplan-Meier analysis.

**Results:**

The CRC patients with the XPD Gln allelic variants had poorer survival than patients with the Lys/Lys genotype (HR  = 1.38, 95% CI  = 1.02–1.87), and rectal cancer patients had the poorest survival among them (HR  = 1.87, 95% CI  = 1.18–2.95). A significantly shorter OS was observed among stage II/III colon cancer patients with the XRCC1 Gln allelic variants (HR  = 1.69, 95% CI  = 1.06–2.71), compared to those with XRCC1 Arg/Arg genotype. In the combined analysis of the XRCC1 and XPD genes patients with stage II/III tumors, the poorest OS occurred in colon cancer patients with the XRCC1 Gln and XPD Gln allelic variants (HR  = 2.60, 95% CI  = 1.19–5.71) and rectal cancer patients with the XRCC1 Arg/Arg and XPD Gln allelic variants (HR  = 2.77, 95% CI  = 1.25–6.17).

**Conclusion:**

The XPD and XRCC1 allelic variants may be prognostic markers for CRC patients receiving 5-FU based chemotherapy. The contributions of the XPD and XRCC1 allelic variants to OS are tumor site- and/or stage-dependent.

## Introduction

After surgery, most metastatic colorectal cancer (CRC) patients receive an adjuvant chemotherapy regimen consisting of combination therapy using 5-fluorouracil (FU) and oxaliplatin (FOLFOX) or leucovorin (LV) for 6 to 8 mo to reduce the probability of tumor recurrence and prolong survival [1]–[3]. The functional single-nucleotide polymorphisms (SNPs) in drug-targeted genes, including xenobiotic-metabolizing and DNA-repair genes, correlate with variability in clinical outcome in multiple types of cancer [4]–[8]. The identification of genetic markers may help identify patients who may benefit from chemotherapy and reduce potential toxicity.

Variable chemosensitivity may involve the detoxification pathway, including the glutathione S-transferases (GSTs). The GSTs are multigene families of enzymes that inactivate electrophilic xenobiotics by conjugation with glutathione, preventing DNA damage and adduct formation [9]. Interindividual differences in GST activity may be mediated by genetic polymorphisms [9]–[12]. The structural deletion polymorphisms in GSTM1 and GSTT1 result in the loss of enzyme-catalyzed detoxification activity [10], and are predictors of clinical outcome in gastric cancer patients receiving platinum/5-FU chemotherapy [13]. In addition, the reduced glutathione conjugation resulting from polymorphism in the GSTP1 Ile105Val (rs1695) coding region may be associated with an increased survival in CRC patients treated with oxaliplatin/5-FU [14], [15].

Various DNA-repair enzymes play important roles in preventing treatment resistance and protecting the genome against carcinogenesis [16]–[19]. The expression of the base excision repair (BER) gene family is triggered by internal oxidative stress and DNA damage [20]. The BER pathway involves the X-ray cross-complementing group 1 gene XRCC1 [21]. The XRCC1 protein directly associates with polymerase beta, DNA ligase III, and poly (ADP-ribose) polymerase (PARP) in single-strand break-repair processes that may play a role in tumor cell sensitivity to 5-FU treatment [22], [23]. The XRCC3 protein, a member of the Rad51-related enzyme family, contributes to the maintenance of chromosome stability through DNA double-strand break/recombination repair in homologous recombination [24].

The nucleotide excision repair (NER) gene family functions within a range of structurally unrelated DNA lesions and DNA adducts [16], [17]. The xeroderma pigmentosum complementation group D (XPD) protein, a member of the NER family functions in sensing, binding, and the subsequent recruitment of repair-related factors [5]. Previous studies have shown that the allelic variants XRCC1 Arg399Gln (rs25487), XRCC3 Thr241Met (rs861539), and XPD Lys751Gln (rs13181) are associated with DNA adduct levels and repair capacity [5], [25], [26]. Therefore, these polymorphisms of DNA-repair genes may affect clinical outcomes in cancer patients receiving chemotherapy [7], [13], [20], [27], [28].

Our previous studies have shown that the null genotypes of GSTM1 and GSTT1, GSTP1 Ile105Val, XRCC1 Arg399Gln, XRCC3 Thr241Met, and XPD Lys751Gln allelic variants are associated with significantly increased risks of CRC [29]–[32]. However, other previous reports of associations between GST and DNA-repair allelic variants and clinical outcomes in CRC have been conflicting [6], [11], [33]–[35]. Whether these conflicting findings have been due to tissue-specific differences in gene expression between colonic and rectal tumors is unclear [36] because reports of stratified analyses based on the site of the CRC tumor are scant. Therefore, in our present study, we investigated the relationship between the allelic variants of relevant GST and DNA-repair genes and the chemotherapeutic outcomes in a retrospective CRC cohort who received adjuvant chemotherapy in Taiwan to determine whether differences exist among the associations in the tumor site or pathological stage.

## Materials and Methods

### Participant selection

Our study was approved by the Institutional Review Board of China Medical University Hospital and Chang Gung Memorial Hospital (CGMH). All patients provided written informed consent before participation in our study. We reviewed 2716 newly diagnosed and histologically confirmed CRC patients who underwent surgery at CGMH between January 1995 and December 2001. We enrolled 499 patients without familial adenomatous polyposis or hereditary nonpolyposis colorectal cancer who underwent 5-FU-based adjuvant chemotherapy as first-line treatment after surgery, and received follow-up examinations every 3 to 6 mo at the outpatient clinic of the Colorectal Section of CGMH.

The postoperative adjuvant chemotherapy regimens included both daily and weekly or monthly treatments. In the daily treatment, tegafur (300–350 mg/m^2^/d) and levamisole (45 mg/d) were administered orally in 3 doses per day for 28 d, followed by a 7-d rest period, totaling 12 mo. The weekly treatments consisted of one of the following treatment schemes for 6 wk, followed by a 2 wk rest, totaling 12 mo: (1) 5-FU (450 mg/m^2^/d) and leucovorin (50 mg/d) were administered as an intravenous bolus once weekly; (2) 5-FU (450 mg/m^2^/d) were administered intravenously once weekly, and levamisole (50 mg) was administered orally 3 times per day for 3 d every 2 wk; or (3) a 24-h intravenous infusion of 5-FU (2600 mg/m^2^/d) and leucovorin (150 mg/d) was administered once weekly. The monthly treatments consisted of one of the following treatment schemes for 6 mo: (1) a continuous 5 d infusion of 5-FU (800 mg/m^2^/d) and leucovorin (50 mg/d) was administered once monthly, or (2) patients received a 5 d continuous infusion of 5-FU (800 mg/ m^2^/d) once monthly combined with levamisole (50 mg) administered orally 3 times per day for 3 d every 2 wk.

### Clinical and questionnaire assessment

The clinical data included a medical history, physical examination (including a rectal and perineal examination), and the measurement of carcinoembryonic antigen (CEA). Liver sonograms, chest X-ray, and colonoscopy (or barium enema examination) were performed annually. All patients were followed until May 2008, with a median follow-up time of 48.8 mo (range  = 1.5 to 133.3 mo). Overall survival (OS) was measured from the date of surgery until death from any cause. In the calculation of OS, the event date represented the date of death, and the censoring date represented the last known date on which the patient was known to be alive.

### DNA extraction and genotyping

The details of the genotyping methods have been described elsewhere [29], [30]. DNA was isolated from peripheral blood leukocytes by using sodium dodecyl sulfate (SDS)-proteinase K-RNase digestion and phenol-chloroform extraction. The GSTM1 and GSTT1 genetic variants were identified using polymerase chain reaction (PCR), and the GSTP1 Ile105Val, XRCC1 Arg399Gln, XRCC3 Thr241Met, and XPD Lys751Gln allelic variants were analyzed using PCR combined with restriction fragment length polymorphism.

The presence or absence of GSTM1, GSTT1, or β-globin (internal control) DNA was detected during multiple PCRs with the following primers: GSTM1 (5′-TGCCCTACTTGATTGATGGG-3′ and 5′-CTGGATTGTAGCAGATCATGC-3′); GSTT1 (5′-TTCCTTACTGGTCCTCACATCTC-3′ and 5′-CACCGGATCATGGCCAGCA-3′); and β-globin (5′-CACAACTGTGTTCACTAGC-3′ and 5′-CAACTTCATCCACGTTCACC-3′). The primer sequences used to detect the genotype corresponding to GSTP1 Ile105Val DNA were 5′-CCTCTCCCTTTCCTCTGTTC-3′ and 5′-CAGGTGAGGGGGACATCT-3. The GSTP1 Ile105Val PCR product was digested using *Alw*26I (New England Biolabs, Beverly, MA, USA). The primer sequences used to detect the genotype corresponding to XRCC1 Arg399Gln DNA were 5′-TTGTGCTTTCTCTGTGTCCA-3′ and 5′-TCCTCCAGCCTTTTCTGATA-3′. The XRCC1 Arg399Gln PCR product was digested using *Msp*I (New England Biolabs). The primer sequences used to detect the genotype corresponding to XRCC3 Thr241Met DNA were 5′-GCCTGGTGGTCATCGACTC-3′, and 5′-ACAGGGCTCTGGAAGGCACTGCTCAGCTCACGCACC-3′. The XRCC3 Thr241Met PCR product was digested using *Nco*I (New England Biolabs). The primer sequences used to detect the genotype corresponding to XPD Lys751Gln DNA were 5′-CCTCTCCCTTTCCTCTGTTC-5′, and 5′-CAGGTGAGGGGGACATCT-3′. The XPD Lys751Gln PCR product was digested using *Pst*I (Takara Bio, Shiga, Japan). For quality control, 10% of the PCRs were randomly repeated, and showed 100% concordance for all the allelic variants analyzed. All laboratory personnel were blinded to the survival status of the patient samples.

### Statistical analysis

We used χ^2^-tests to evaluate possible associations between the categorical variables, and continuous variables were analyzed using a Student *t* test. Allelic frequencies were compared with those expected at the Hardy-Weinberg equilibrium (HWE) by using a χ^2^ test. Univariate analysis of the Kaplan-Meier estimates and the log-rank test were used to compare the overall survival curves. A Cox proportional-hazards model was used to estimate the hazard ratios (HRs) and the 95% confidence intervals (CIs) for the various allelic variants associated with OS. Age, sex, and the tumor, node, metastasis (TNM) stage were considered potential confounding covariates, and were thus included in the multivariate regression. The Cox proportional-hazards regression model was based on a priori knowledge of factors known to carry prognostic or predictive information to estimate adjusted HRs and 95% CIs. The HRs for differences in OS based on the tumor site and stage were estimated using stratified analysis. All models were examined for adherence to the proportional-hazards assumption by assessing the log-minus-log survival plots and performing the Schoenfeld test. The log-minus-log survival plots and the results of the Schoenfeld tests (*P* range  = 0.22 to 0.90) indicated no violations of the proportionality assumption for the 6 SNPs investigated. All analyses were performed using the SAS statistical software package, version 9.3 for Windows (SAS Institute, Cary, NC, USA). The results of comparisons with a 2 sided *P* value less than 0.05 were considered to represent statistically significant relationships. To account for multiple comparisons, we calculated the false discovery rate (FDR) by using the PROC MULTTEST in SAS. The statistical power of our analyses was estimated using the PASS statistical software package, version 11 (NCSS, Kaysville, UT, USA).

## Results

### Demographic and clinical characteristics

Eight CRC patients who failed genotyping were excluded from further analysis. The demographic and clinical characteristics of the 491 patients are summarized in Table 1. The mean age and standard deviation (SD) was 58.5±12.5 y. Our cohort consisted of 260 (52.9%) men, and 168 (35.5%) patients had a family history of cancer. The primary tumor was located in the colon in 283 (57.6%) patients and in the rectum in 208 (42.4%) patients. Multiple primary malignancies occurred in 4.9% of the cohort, and 78.4% had a moderately differentiated tumor. The CEA was present at ≥5 ng/mL in 53.6% of the cohort. In the TNM evaluation, 74 (15.1%), 267 (54.4%), and 150 (30.5%) patients were classified as being stage II, III, and IV, respectively. Compared to patients with rectal cancer, colon cancer patients were younger, were diagnosed more frequently with multiple malignancies, presented more often with moderately differentiated tumors, and were less likely to be TNM stage III (*P*<0.05). No significant associations were found between sex, family history of cancer, or CEA level and the tumor site.

**Table 1:** Demographic characteristics, clinical features, and allele distributions of study patients.

	Total	Colon	Rectum	
Variables^a^	N (%)	N (%)	N (%)	*P*
Age^b^ (y)	58.5±12.5	57.1±12.9	60.4±11.6	0.004^c^
Sex				0.105^d^
Male	260 (52.9)	141 (49.8)	119 (57.2)	
Female	231 (47.1)	142 (50.2)	89 (42.8)	
Family history of cancer				0.961^d^
No	305 (64.5)	175 (64.6)	130 (64.4)	
Yes	168 (35.5)	96 (35.4)	72 (35.6)	
Multiplicity				0.029^d^
No	467 (95.1)	264 (93.3)	203 (97.6)	
Yes	24 (4.9)	19 (6.7)	5 (2.4)	
Histological differentiation				0.009^d^
Well	59 (12.1)	38 (13.6)	21 (10.1)	
Moderately	382 (78.4)	207 (73.9)	175 (84.5)	
Poorly	46 (9.5)	35 (12.5)	11 (5.4)	
Carcinoembryonic antigen				0.086^d^
<5 ng/mL	219 (46.4)	117 (43.0)	102 (51.0)	
≥5 ng/mL	253 (53.6)	155 (57.0)	98 (49.0)	
TNM stage				0.001^d^
II	74 (15.1)	56 (19.8)	18 (8.7)	
III	267 (54.4)	138 (48.8)	129 (62.0)	
IV	150 (30.5)	89 (31.4)	61 (29.3)	
GSTM1				0.254^d^
Null	286 (58.2)	171 (60.4)	115 (55.3)	
Present	205 (41.8)	112 (39.6)	93 (44.7)	
GSTT1				0.317^d^
Null	249 (50.7)	149 (52.6)	100 (48.1)	
Present	242 (49.3)	134 (47.4)	108 (51.9)	
GSTP1 Ile105Val				0.632^d^
Ile/Ile	336 (68.4)	191 (67.5)	145 (69.7)	
Ile/Val	139 (28.3)	81 (28.6)	58 (27.9)	
Val/Val	16 (3.3)	11 (3.9)	5 (2.4)	
XRCC1 Arg399Gln				0.016^d^
Arg/Arg	249 (50.7)	159 (56.2)	90 (43.3)	
Arg/Gln	212 (43.2)	110 (38.9)	102 (49.0)	
Gln/Gln	30 (6.1)	14 (5.0)	16 (7.7)	
XRCC3 Thr241Met				0.565^e^
Thr/Thr	459 (93.5)	263 (92.9)	196 (94.2)	
Thr/Met	32 (6.5)	20 (7.1)	12 (5.8)	
Met/Met	0 (0.0)	0 (0.0)	0 (0.0)	
XPD Lys751Gln				0.327^e^
Lys/Lys	404 (82.3)	234 (82.7)	170 (81.7)	
Lys/Gln	84 (17.1)	46 (16.2)	38 (18.3)	
Gln/Gln	3 (0.6)	3 (1.1)	0 (0.0)	

a Sum may not be equal to the total number (N) because of missing data.

b Mean±standard deviation.

c Student t-test.

d Chi-squared test.

e Fisher exact test.

TNM: tumor-node-metastasis.

### Genotype frequencies

The genotype distributions of the GST and DNA-repair allelic variants in the CRC patients are also shown in Table 1. The frequencies of the null genotypes of GSTM1 and GSTT1 and the GSTP1 Val, XRCC1 Gln, XRCC3 Met, and XPD Gln allelic variants were 58.2%, 50.7%, 17.4%, 27.7%, 3.3%, and 9.2%, respectively. The allele frequencies of the GSTP1, XRCC1, XRCC3, and XPD allelic variants were at HWE. We found that the distribution of these allelic variants did not vary significantly according to the tumor site, except for the XRCC1 Gln allelic variants (*P* = 0.016). The XRCC1 Arg/Arg genotype was more prevalent in colon cancer patients (56.2%) than in rectal cancer patients (43.3%).

### Association between GSTs and DNA-repair allelic variants and survival

Univariate analysis revealed that the association between the XRCC1 Gln allelic variants and reduced OS was more significant than that of XRCC1 Arg/Arg genotype, with a median survival of 43.0 versus 57.1 mo, respectively (HR  = 1.36, 95% CI  = 1.07–1.73). However, this association was absent following adjustment for the covariates, which included age, sex, and the stage of disease. The other allelic variants analyzed were not associated with OS in univariate analysis of the Kaplan-Meier estimates (data not shown).

Table 2 shows the adjusted associations between the allelic variants and OS in the multivariate Cox proportional-hazards regression model. Among the 6 allelic variants tracked, reduced OS was associated with heterozygous carriers of XPD Lys/Gln (HR  = 1.34, 95% CI  = 0.99–1.84, *P* = 0.062, FDR  = 0.372) and homozygous carriers of XPD Gln/Gln (HR  = 2.38, 95% CI  = 0.75–7.53, *P* = 0.141). Compared to patients with XPD Lys/Lys genotype, patients with XPD Gln allelic variants were significantly associated with reduced OS, with a median survival of 49.0 versus 47.1 mo, respectively (HR  = 1.38, 95% CI  = 1.02–1.88, *P* = 0.039, FDR  = 0.372).

**Table 2:** Cox proportional–hazards analysis of associations between allelic variants of metabolizing and DNA–repair genes and overall survival status among colorectal cancer patients.

Variables	Patients	Events	MSM	HR (95% CI)^a^	*P*	FDR
GSTM1						
Null	286	161	48.1	1 (reference)		
Present	205	107	50.1	0.81 (0.64–1.04)	0.100	0.400
GSTT1						
Null	249	133	49.5	1 (reference)		
Present	242	135	47.6	1.15 (0.90–1.46)	0.271	0.643
GSTP1 Ile105Val						
Ile/Ile	336	183	48.8	1 (reference)		
Ile/Val	139	76	49.0	1.10 (0.84–1.44)	0.482	0.643
Val/Val	16	9	38.9	1.12 (0.57–2.20)	0.749	0.850
Ile/Val+ Val/Val	155	85	49.0	1.10 (0.85–1.43)	0.459	0.643
XRCC1 Arg399Gln						
Arg/Arg	249	126	57.1	1 (reference)		
Arg/Gln	212	126	42.3	1.12 (0.87–1.44)	0.389	0.643
Gln/Gln	30	16	49.0	1.06 (0.63–1.79)	0.827	0.850
Arg/Gln + Gln/Gln	242	142	43.0	1.11 (0.87–1.42)	0.402	0643
XRCC3 Thr241Met						
Thr/Thr	459	253	48.6	1 (reference)		
Thr/Met	32	15	49.3	1.05 (0.62–1.78)	0.850	0.850
Met/Met	0	0	0	–	–	–
XPD Lys751Gln						
Lys/Lys	404	216	49.0	1 (reference)		
Lys/Gln	84	49	47.8	1.34 (0.99–1.84)	0.062	0.372
Gln/Gln	3	3	28.5	2.38 (0.75–7.53)	0.141	0.423
Lys/Gln + Gln/Gln	87	52	47.1	1.38 (1.02–1.88)	0.039	0.372

a Adjusted for age, sex, and tumor-node-metastasis stage.

MSM: median survival month; FDR: false discovery rate.

### Association between the genotype and survival by tumor site and TNM stage

The results of the stratified analyses of OS associated with the GSTs and DNA-repair allelic variants based on the tumor location are shown in Table 3. Rectal cancer patients with the XPD Gln allelic variants had a shorter OS compared with patients with XPD Lys/Lys genotype, with a median survival 44.9 versus 51.6 mo (log-rank test *P* = 0.230). The multivariate Cox regression model also showed that the XPD Gln allelic variants were associated with an 87% increased risk of shorter OS compared to XPD Lys/Lys genotype (HR  = 1.87; 95% CI  = 1.18–2.95, *P* = 0.007, FDR  = 0.084). However, no adverse effect of Gln allelic variants on OS was observed in colon cancer patients. No significant association between the other allelic variants and OS was observed based on the tumor site.

**Table 3:** Cox proportional-hazards analysis of associations between allelic variants and overall survival status among colorectal cancer patients based on tumor location.

	Colon						Rectum					
Variables	Patients	Events	MSM	HR (95% CI)^a^	*P*	FDR	Patients	Events	MSM	HR (95% CI)^a^	*P*	FDR
GSTM1												
Null	171	95	48.1	1 (reference)			115	66	48.5	1 (reference)		
Present	112	57	48.9	0.83 (0.59–1.15)	0.262	0.689	93	50	56.7	0.82 (0.56–1.19)	0.287	0.689
GSTT1												
Null	149	79	49.1	1 (reference)			100	54	55.8	1 (reference)		
Present	134	73	47.1	1.21 (0.88–1.68)	0.240	0.689	108	62	48.5	1.12 (0.77–1.62)	0.559	0.917
GSTP1 Ile105Val												
Ile/Ile	191	102	48.1	1 (reference)			145	81	49.5	1 (reference)		
Ile/Val + Val/Val	92	50	48.6	1.12 (0.80–1.58)	0.506	0.917	63	35	49.0	1.07 (0.72–1.59)	0.745	0.917
XRCC1 Arg399Gln												
Arg/Arg	159	75	57.1	1 (reference)			90	51	57.2	1 (reference)		
Arg/Gln + Gln/Gln	124	77	36.4	1.25 (0.90–1.73)	0.181	0.689	118	65	47.7	0.98 (0.68–1.42)	0.910	0.917
XRCC3 Thr241Met												
Thr/Thr	263	142	48.1	1 (reference)			196	111	49.0	1 (reference)		
Thr/Met	20	10	49.0	1.06 (0.56–2.01)	0.866	0.917	12	5	67.4	0.95 (0.38–2.39)	0.917	0.917
XPD Lys751Gln												
Lys/Lys	234	125	48.0	1 (reference)			170	91	51.6	1 (reference)		
Lys/Gln + Gln/Gln	49	27	49.1	1.10 (0.72–1.67)	0.667	0.917	38	25	44.9	1.87 (1.18–2.95)	0.007	0.084

a Adjusted for age, sex, and tumor-node-metastasis stage.

MSM: median survival month; HR: hazard ratio; CI: confidence interval; FDR: false discovery rate.

The stratified analyses of the associations between OS and the polymorphisms of the GST and DNA-repair alleles based on the TNM stage showed that both stage II/III and stage IV CRC patients with the XPD Gln allelic variants didn't have a significantly poorer OS, with HRs of 1.43 (*P* = 0.085) and 1.31 (*P* = 0.264), respectively, compared to patients with XPD Lys/Lys genotype (Table 4). The association between the XRCC1 Gln allelic variants and reduced OS in stage II/III patients approached significance (HR  = 1.41, 95% CI  = 1.00–1.99, *P*  = 0.051, FDR  = 0.510). The other allelic variants were not associated with OS in stage IV patients.

**Table 4:** Cox proportional-hazards analysis of associations between allelic variants and overall survival status among colorectal cancer patients based on tumor-node-metastasis stage.

	Stage II/III						Stage IV					
Variables	Patients	Events	MSM	HR (95% CI)^a^	*P*	FDR	Patients	Events	MSM	HR (95% CI)^a^	*P*	FDR
GSTM1												
Null	199	79	59.0	1 (reference)			87	82	18.7	1 (reference)		
Present	141	49	60.4	0.80 (0.56–1.15)	0.224	0.713	64	58	20.0	0.87 (0.62–1.22)	0.416	0.713
GSTT1												
Null	176	65	60.6	1 (reference)			73	68	20.2	1 (reference)		
Present	164	63	59.1	1.11 (0.78–1.57)	0.563	0.831	78	72	18.8	1.09 (0.78–1.52)	0.623	0.831
GSTP1 Ile105Val												
Ile/Ile	232	86	60.8	1 (reference)			104	97	19.2	1 (reference)		
Ile/Val + Val/Val	108	42	58.9	1.17 (0.81–1.70)	0.404	0.713	47	43	19.9	1.00 (0.70–1.43)	0.992	0.992
XRCC1 Arg399Gln												
Arg/Arg	180	62	60.9	1 (reference)			69	64	20.0	1 (reference)		
Arg/Gln + Gln/Gln	160	66	56.7	1.41 (1.00–2.00)	0.051	0.510	82	76	18.8	1.02 (0.72–1.43)	0.930	0.992
XRCC3 Thr241Met												
Thr/Thr	314	119	60.0	1 (reference)			145	134	19.4	1 (reference)		
Thr/Met	26	9	67.4	0.91 (0.46–1.79)	0.780	0.936	6	6	17.7	1.42 (0.62–3.27)	0.404	0.713
XPD Lys751Gln												
Lys/Lys	275	97	60.3	1 (reference)			129	119	19.6	1 (reference)		
Lys/Gln + Gln/Gln	65	31	58.6	1.43 (0.95–2.14)	0.085	0.510	22	21	17.7	1.31 (0.82–2.09)	0.264	0.713

a Adjusted for age and sex.

MSM: median survival month; HR: hazard ratio; CI: confidence interval; FDR: false discovery rate.

Table 5 shows the associations of the XRCC1 and XPD allelic variants with OS in CRC patients stratified by tumor site and TNM stage. The poorest OS was observed among the stage II/III colon cancer patients with the XRCC1 Gln allelic variants (HR  = 1.69, 95% CI  = 1.06–2.71, *P*  = 0.028, FDR  = 0.252). Although the rectal cancer patients who inherited XPD Gln allelic variants had significantly reduced OS, this negative effect was not more prominent in any specific TNM stage (stage II/III, HR  = 1.57, 95% CI  = 0.88–2.81; stage IV, HR  = 1.98, 95% CI  = 0.94–4.17).

**Table 5:** Tumor site- and tumor-node-metastasis stage-specific hazard ratios for the associations between the XRCC1 Arg399Gln and XPD Lys751Gln allelic variants and overall survival among colorectal cancer patients.

	Colon						Rectum					
	Stage II/III			Stage IV			Stage II/III			Stage IV		
Variables	HR (95% CI)^a^	*P*	FDR	HR (95% CI)^a^	*P*	FDR	HR (95% CI)^a^	*P*	FDR	HR (95% CI)^a^	*P*	FDR
XRCC1 Arg399Gln												
Arg/Gln+Gln/Gln vs Arg/Arg	1.69 (1.06–2.71)	0.028	0.252	1.05 (0.67–1.66)	0.829	0.933	1.15 (0.67–1.96)	0.612	0.918	0.93 (0.55–1.59)	0.794	0.933
XPD Lys751Gln												
Lys/Gln+Gln/Gln vs Lys/Lys	1.28 (0.72–2.26)	0.403	0.727	1.00 (0.54–1.85)	0.999	0.999	1.57 (0.88–2.81)	0.125	0.375	1.98 (0.94–4.17)	0.072	0.324

a Adjusted for age and sex.

HR: hazard ratio; CI: confidence interval; FDR: false discovery rate.

**Figure 1:**
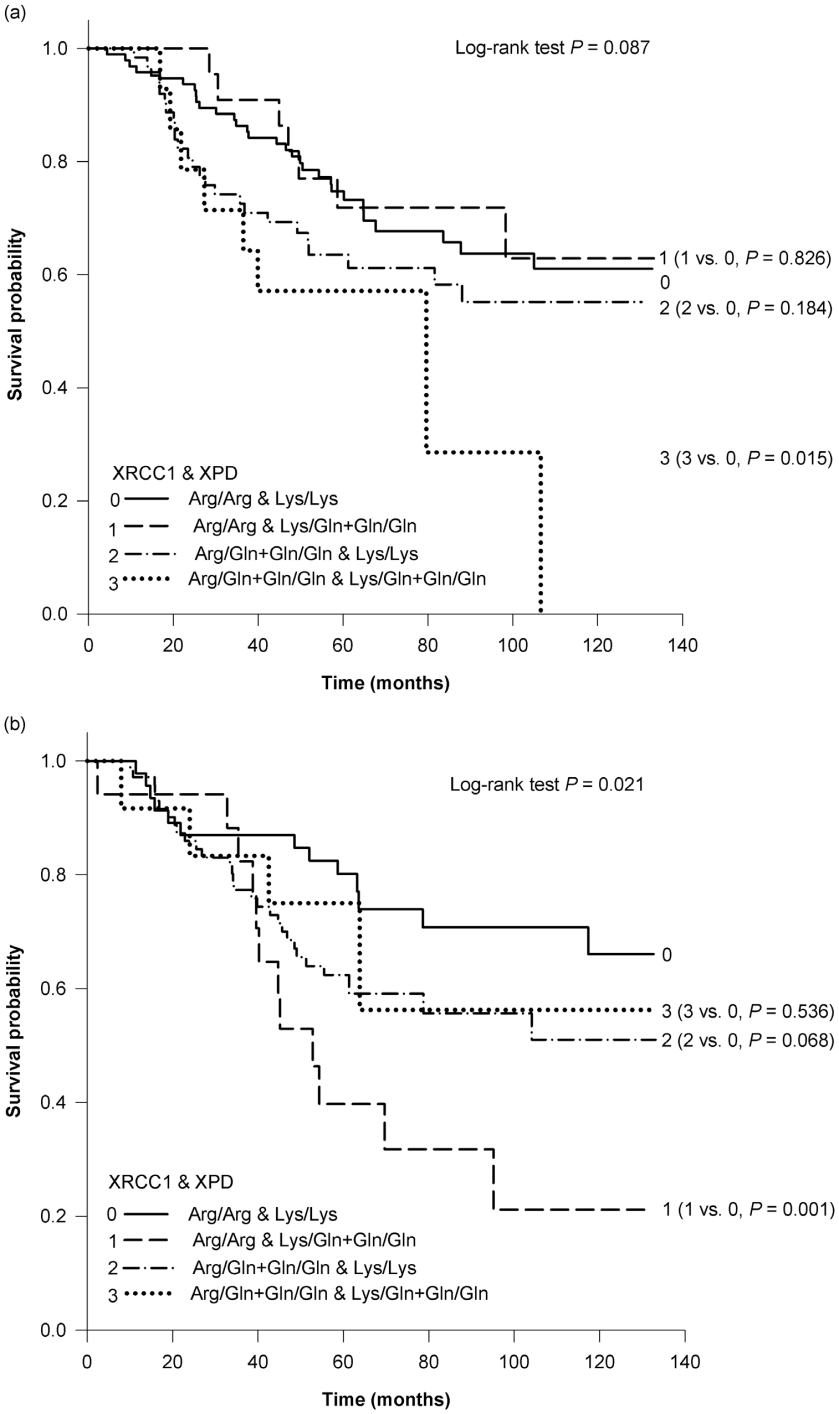
Kaplan-Meier estimates of the effect of coexisting XRCC1 and XPD allelic variants on overall survival in colorectal cancer patients stratified by tumor site and tumor-node-metastasis stage. (a) The survival curve of stage II/III colon cancer patients (overall log-rank test *P* = 0.087). (b) The survival curve of stage II/III rectal cancer patients (overall log-rank test *P* = 0.021).

In the combined analysis of the XRCC1 and XPD allelic variants, the Kaplan-Meier survival curves showed differences in OS among the 4 allelic variants analyzed in stage II/III colon cancer patients (log-rank test *P* = 0.087, Figure 1a). Compared to patients with XRCC1 Arg/Arg and XPD Lys/Lys genotype, those with XRCC1 Gln and XPD Gln allelic variants had poorer OS (log-rank test *P* = 0.015), with an HR of 2.60 (95% CI  = 1.19–5.71, FDR  = 0.102, Table 6). Kaplan-Meier analysis showed significant variability in survival (log-rank test *P* = 0.021) among the subgroups in stage II/III rectal cancer patients (Figure 1b). The XRCC1 Arg/Arg and XPD Gln allelic variants were associated with a significantly poorer OS, compared with that of patients with XRCC1 Arg/Arg and XPD Lys/Lys genotype (log-rank test *P* = 0.001; HR  = 2.77, 95% CI, 1.25–6.17, FDR  = 0.102). However, this effect on OS was not observed among stage IV colon or rectal cancer patients with the XRCC1 Arg/Arg and XPD Gln allelic variants. FDR analysis indicated that the combined HRs of the XRCC1 Gln and XPD Gln allelic variants in stage II/III colon cancer patients (HR  = 2.60; FDR  = 0.102) and the XRCC1 Arg/Arg and XPD Gln allelic variants in stage II/III rectal cancer patients (HR  = 2.77; FDR  = 0.102) were not significantly influenced by type I error because of multiple comparisons. However, the statistical power of our analysis was determined to be 63% (HR  = 2.60) and 49% (HR  = 2.77) for these associations.

**Table 6:** Tumor site- and tumor-node-metastasis stage-specific hazard ratios for the associations between the coexisting XRCC1 Arg399Gln and XPD Lys751Gln allelic variants and overall survival among colorectal cancer patients.

		Colon						Rectum					
		Stage II/III			Stage IV			Stage II/III			Stage IV		
XRCC1	XPD	HR (95% CI)^a^	*P*	FDR	HR (95% CI)^a^	*P*	FDR	HR (95% CI)^a^	*P*	FDR	HR (95% CI)^a^	*P*	FDR
Arg/Arg	Lys/Lys	1 (reference)			1 (reference)			1 (reference)			1 (reference)		
Arg/Arg	Lys/Gln + Gln/Gln	1.03 (0.45–2.35)	0.945	0.967	0.98 (0.41–2.38)	0.967	0.967	2.77 (1.25–6.17)	0.012	0.102	1.63 (0.59–4.46)	0.345	0.690
Arg/Gln + Gln/Gln	Lys/Lys	1.54 (0.90–2.62)	0.115	0.324	1.05 (0.64–1.72)	0.863	0.967	1.73 (0.88–3.38)	0.110	0.324	0.91 (0.50–1.64)	0.744	0.967
Arg/Gln + Gln/Gln	Lys/Gln + Gln/Gln	2.60 (1.19–5.71)	0.017	0.102	1.07 (0.44–2.56)	0.885	0.967	1.43 (0.46–4.44)	0.535	0.917	2.40 (0.76–7.59)	0.135	0.324

a Adjusted for age and sex.

HR: hazard ratio; CI: confidence interval; FDR: false discovery rate.

## Discussion

We found a significant relationship between the XPD Lys751Gln allelic variant and OS for CRC patients, particularly for rectal cancer patients, whereas XRCC1 Arg399Gln Gln allele correlated with reduced OS in stage II/III colon cancer patients. In addition, the poorest OS was present among the stage II/III colon cancer patients with both XRCC1 Gln and XPD Gln allelic variants and among the stage II/III rectal cancer patients with both the XRCC1 Arg/Arg and XPD Gln allelic variants. However, significant associations were not observed between OS and the other GST and DNA-repair gene allelic variants in CRC patients receiving 5-FU-based chemotherapy.

The XPD gene is an important DNA-repair gene that codes for enzymes that recruit NER in the repair of a wide range of DNA lesions [16], [17]. Mutations that alter the amino acid sequence impact the interactions of XPD enzyme with other members, such as XPA, ERCC1, and replication protein A of the NER complex, resulting in different DNA-repair activities [18]. The XPD Lys751Gln polymorphism converts the basic amino acid, Lys, to the polar amino acid, Gln, at approximately 50 bp upstream from the poly(A) signal, which may affect the function of the XPD protein [4]. Patients with the XPD Lys/Lys genotype have sub-optimal DNA-repair activity, and are more sensitive to chemotherapy [5], [6].

Previous studies have shown that the XPD Lys751Gln allelic variant may be associated with various clinical outcomes in CRC patients receiving chemotherapy [6], [24], [34], [37]. Park et al [6] found that advanced-stage CRC patients with XPD Gln/Gln genotype treated with chemotherapy tended to have progressive disease and significantly reduced survival, compared to patients with XPD Lys allelic variants (*P* = 0.002). A previous study of XPD Lys751Gln in 166 advanced CRC patients receiving FOLFOX therapy found a negative relationship between XPD Gln/Gln genotype and adverse progression-free survival (HR  = 2.21, 95% CI  = 1.17–4.17, *P*  = 0.01) [34]. Our results are consistent with previous studies that have shown a negative association between the XPD Gln allelic variants and OS. However, 2 studies have reported no significant relationship between XPD Lys751Gln and clinical outcomes in CRC patients [7], [33].

We demonstrated that the reduction in OS associated with XPD Lys751Gln Gln allelic variants were improved in rectal cancer patients. In the contrast, Duldulao et al. [38] observed that XPD Lys/Lys genotype was significantly associated with increased toxicity to neoadjuvant chemoradiotherapy in 132 stage II/III rectal cancer patients in the United States. However, Cecchin et al [39] reported no association between the XPD Lys751Gln allelic variant and the tumor regression grade in 238 rectal cancer patients treated with neoadjuvant chemoradiotherapy in Italy. Different ethnicities, treatment regiments, and outcome measurements may have contributed to these inconsistencies.

The association between the XRCC1 Arg399Gln allelic variant and OS has been previously reported [35], [37]. Artac et al [37] recruited 43 metastatic CRC patients who received irinotecan-based therapy, and found that OS is associated with XRCC1 Gln/Gln genotype (HR  = 2.85, *P* = 0.04). In a study of stage II/III rectal cancer patients in Spain, patients with XRCC1 Arg/Arg had a greater probability of a positive response to chemoradiotherapy than those with XRCC1 Arg/Gln (OR  = 4.18, 95% CI  = 1.62–10.7) [35]. In our study, we observed that only the XRCC1 Gln allelic variants were significantly associated with reduced OS in stage II/III colon cancer patients.

In contrast, Huang et al [22] showed that XRCC1 Gln/Gln genotype was significantly associated with favorable OS (HR  = 0.15, 95% CI  = 0.04–0.57) and progression-free survival (HR  = 0.31, 95% CI  = 0.10–0.91) in metastatic CRC patients receiving FOLFOX-4 chemotherapy. Previous reports have suggested that the XRCC1 Gln allelic variants are deficient in DNA-repair activity, leading to increased chromosomal damage [27], [40], [41]. Thus, suboptimal repair activity in tissues favors carcinogenesis, but may ensure tumor sensitivity to drug or ionizing treatment [42]. However, other studies have shown that the XRCC1 Arg399Gln allelic variant was not significantly associated with the outcome [7], [22] or response to chemotherapy [33], [34], [43].

In our analysis of the associations between the allelic variants of GST and DNA-repair genes and the OS stratified by tumor site and stage, we found that the XPD 751Gln and the XRCC1 399Gln allelic variants were significantly associated with reduced OS for stage II/III rectal cancer and colon cancer, respectively. Although previous studies have not shown that specific DNA repair activity may vary between the colon and the rectum, a high proportion of rectal tumors has been shown to have reduced levels of thymidylate synthase, based on enzyme activity assays [44], [45]. Despite the relatively small number of cases and the lack of statistical significance for the results, our findings support an association between polymorphisms in DNA-repair genes and OS based on specific tumor locations among CRC patients treated with 5-FU chemotherapy. The prognostic value of variability in DNA-repair activity based on a CRC tumor site warrants further study.

Similar to the findings of previous studies, we found no association between OS and the XRCC3 Thr241Met, GSTM1, GSTT1, and GSTP1 Ile105Val allelic variants [14], [35], [39], [46]. A meta-analysis of 13 independent studies that included 1234 advanced or metastatic CRC patients found no significant association between the GSTP1 Ile105Val allelic variant and tumor response [46]. However, Stoehlmacher et al [11] observed that GSTP1 Val/Val genotype was associated with increased survival in CRC patients following combination therapy with oxaliplatin and 5-FU. The association between the GSTP1 Ile105Val allelic variant and the chemotherapeutic outcome in CRC also warrants further study.

There are potential limitations to our findings. First, all of our participants received 5-FU-based chemotherapy. Thus, we did not investigate the genotypes associated with clinical outcomes in an untreated control group. Second, we did not examine mRNA expression or perform immunohistochemistry analysis of tumor tissues. Therefore, our analysis was not free of potential biases, and does not account for the loss of heterozygosity in the tumor. However, immunohistochemistry is a subjectively semiquantitative method, and is limited by the sensitivity of the antibodies and tissue-handling techniques used. Third, our study design lacked statistical power to support the significance of the association between the XPD Ly751Gln and XRCC1 Arg399Gln allelic variants and the OS in CRC patients treated with 5-FU-based chemotherapy (49% and 63%, respectively). Nevertheless, our study included newly diagnosed and histologically confirmed CRC patients, a relatively large sample, and a long-term follow-up. Thus, our results may shed light on the value of the XPD Ly751Gln and XRCC1 Arg399Gln allelic variants as prognostic markers for CRC of various tumor sites and stages.

In conclusion, we evaluated multiple xenobiotic-metabolizing and DNA-repair genetic polymorphisms as prognosticators of OS in CRC patients receiving 5-FU-based chemotherapy. Our results showed that the XPD and XRCC1 allelic variants were tumor site- and/or stage-dependently associated with OS for CRC patients receiving 5-FU-based chemotherapy. Further studies are warranted to identify the underlying biochemical mechanisms affected by the mutations in the allelic variants, and to validate the roles of XPD and XRCC1 genetic polymorphisms as predictors of chemotherapeutic outcome in CRC patients.
