# Correction: Tumor Site- and Stage-Specific Associations between Allelic Variants of Glutathione S-Transferase and DNA-Repair Genes and Overall Survival in Colorectal Cancer Patients Receiving 5-Fluorouracil-Based Chemotherapy

**DOI:** 10.1371/annotation/6f8b9855-762b-4011-bd03-79bb868607db

**Published:** 2013-07-31

**Authors:** Ching-Yu Lai, Ling-Ling Hsieh, Fung-Chang Sung, Reiping Tang, Chyi-Huey Bai, Fang-Yang Wu, Hung-Yi Chiou, Chih-Ching Yeh

The number of the grant from Taipei Medical University (beginning "TMU...") is incorrect. The correct grant number is: TMU99-AE1-B11.

